# The Multidrug Efflux Regulator AcrR of Escherichia coli Responds to Exogenous and Endogenous Ligands To Regulate Efflux and Detoxification

**DOI:** 10.1128/msphere.00474-22

**Published:** 2022-11-23

**Authors:** Dana E. Harmon, Cristian Ruiz

**Affiliations:** a Department of Biology, California State University Northridge, Northridge, California, USA; Antimicrobial Development Specialists, LLC

**Keywords:** AcrR, ligand, polyamines, ethidium bromide, multidrug efflux pump, AcrAB-TolC, *Escherichia coli*

## Abstract

The transcriptional repressor AcrR is the main regulator of the multidrug efflux pump AcrAB-TolC, which plays a major role in antibiotic resistance and cell physiology in Escherichia coli and other *Enterobacteriaceae*. However, it remains unknown which ligands control the function of AcrR. To address this gap in knowledge, this study tested whether exogenous and/or endogenous molecules identified as potential AcrR ligands regulate the activity of AcrR. Using electrophoretic mobility shift assays (EMSAs) with purified AcrR and the *acrAB* promoter and *in vivo* gene expression experiments, we found that AcrR responds to both exogenous molecules and cellular metabolites produced by E. coli. In total, we identified four functional ligands of AcrR, ethidium bromide (EtBr), an exogenous antimicrobial known to be effluxed by the AcrAB-TolC pump and previously shown to bind to AcrR, and three polyamines produced by E. coli, namely, putrescine, cadaverine, and spermidine. We found that EtBr and polyamines bind to AcrR both *in vitro* and *in vivo*, which prevents the binding of AcrR to the *acrAB* promoter and, ultimately, induces the expression of *acrAB*. Finally, we also found that AcrR contributes to mitigating the toxicity produced by excess polyamines by directly regulating the expression of AcrAB-TolC and two previously unknown AcrR targets, the MdtJI spermidine efflux pump and the putrescine degradation enzyme PuuA. Overall, these findings significantly expand our understanding of the function of AcrR by revealing that this regulator responds to different exogenous and endogenous ligands to regulate the expression of multiple genes involved in efflux and detoxification.

**IMPORTANCE** Multidrug efflux pumps can remove antibiotics and other toxic molecules from cells and are major contributors to antibiotic resistance and bacterial physiology. Therefore, it is essential to better understand their function and regulation. AcrAB-TolC is the main multidrug efflux pump in the *Enterobacteriaceae* family, and AcrR is its major transcriptional regulator. However, little is known about which ligands control the function of AcrR or which other genes are controlled by this regulator. This study contributes to addressing these gaps in knowledge by showing that (i) the activity of AcrR is controlled by the antimicrobial ethidium bromide and by polyamines produced by E. coli, and (ii) AcrR directly regulates the expression of AcrAB-TolC and genes involved in detoxification and efflux of excess polyamines. These findings significantly advance our understanding of the biological role of AcrR by identifying four ligands that control its function and two novel targets of this regulator.

## INTRODUCTION

Antibiotic resistance is one of the most significant threats to public health worldwide (1, 2). Multidrug efflux (MDR) pumps are intrinsically present in all bacteria and are major contributors to antibiotic resistance and virulence, especially in Gram-negatives (3–7). MDR pumps can expel many structurally unrelated antibiotics and other toxic compounds (3–5), acting synergistically with the permeability barrier provided by the outer membrane of Gram-negatives to prevent the accumulation of antibiotics (3–5). Moreover, MDR pumps play an increasingly recognized role in bacterial metabolism and physiology (3–5, 8–15). Under laboratory conditions, AcrAB-TolC is the main MDR pump in Escherichia coli and other *Enterobacteriaceae* (3–5). Its expression is directly controlled by several transcriptional regulators, including the activators MarA, SoxS, and Rob, and the AcrR repressor (3–5, 11, 16–20). Of them, AcrR is the main regulator of AcrAB-TolC expression by directly regulating *acrAB* (11, 16) and *marRAB* and *SoxRS* (21), which encode the *acrAB* and *tolC* transcriptional regulators MarA and SoxS. AcrR also regulates its own expression (16).

The *acrR* gene is located 141 bp upstream of the *acrAB* operon and is divergently transcribed (16). AcrR is a 24.8-kDa protein that belongs TetR family of transcriptional regulators, which are characterized by a conserved three-helix N-terminal DNA-binding domain and a diverse C-terminal ligand-binding domain (22–24). However, despite its central role in regulating the expression of the AcrAB-TolC pump, there is little knowledge about which ligands control the activity of AcrR.

To date, only two studies have investigated this question. First, Su et al. (25) used fluorescence polarization assays to determine that three antimicrobials known to be substrates of the AcrAB-TolC pump, ethidium bromide, proflavine, and rhodamine 6G (3–5), bind to purified AcrR with a similar dissociation constant to that previously found for AcrB (26). Second, Li et al. (23) determined the crystal structure of AcrR and predicted its large ligand-binding pocket computationally. The authors then identified a key amino acid within the pocket, E67, and demonstrated by fluorescence polarization that the E67A mutant was no longer able to bind these three compounds with high affinity (23). However, when they used docking analysis to further study the binding of these compounds to AcrR, the authors found that ethidium bromide and proflavine, but not rhodamine 6G, fit in the ligand-binding pocket (23). Moreover, despite these findings, it remains unknown whether binding of any of these exogenous antimicrobial ligands to AcrR translates into an altered binding of this regulator to DNA and, thus, into changes in gene expression of AcrR-regulated genes.

A second major gap in knowledge is determining whether AcrR also responds to endogenous ligands. Several studies support the hypothesis that endogenous ligands and, in particular, endogenous AcrAB-TolC substrates, regulate the activity of AcrR. First, using Δ*acrA*, Δ*acrB*, and Δ*tolC* knockout mutants or the efflux pump inhibitor phenylalanine-arginine-β-naphthylamide, Ruiz and Levy (11) found that the AcrAB-TolC pump of E. coli plays a major role in regulating its own expression. Moreover, this study revealed that upregulation of the *acrAB* promoter in the Δ*acrB* mutant was mediated by the accumulation of cellular metabolites that changed the activity of AcrR and, to a lesser degree, the expression of MarA and SoxS (11). Ruiz and Levy also found that deletion of *acrB* altered the expression of many metabolic genes, strongly upregulated the expression of motility genes, and increased motility in this mutant (11).

Second, the connection between AcrAB-TolC function, metabolism, motility, and AcrR activity is further supported by a later study by Kim et al. (27). They showed that deletion of *acrR* in E. coli altered the expression of many metabolic genes, upregulated the expression of motility genes, and increased motility (27) in a similar manner as previously observed in the Δ*acrB* mutant (11). Given that AcrR represses *acrAB* transcription, this finding might seem counterintuitive. However, we hypothesize that the similar gene expression and motility changes found in both mutants may occur because cellular metabolites that accumulate in the Δ*acrB* mutant can bind to and inactivate AcrR. Such metabolite inactivation of AcrR would explain why the Δ*acrB* mutant behaved as the Δ*acrR* mutant in these studies (11, 27).

Finally, this model is further supported by a study from our lab (12) in which we studied Δ*acrB*, Δ*tolC*, and Δ*acrR* E. coli mutants using untargeted metabolomics and identified global changes in the intracellular and extracellular metabolite profiles in all three mutants compared to the parental strain. These findings and the studies discussed above suggest that the cellular metabolites found to most significantly accumulate in the Δ*acrB* mutant (12), or their metabolic precursors or derivatives, may actively be effluxed by the AcrAB-TolC pump. Moreover, we also hypothesize that some of these cellular metabolites may regulate the activity of AcrR, the main repressor of the *acrAB* operon.

Here, we show that the antimicrobial ethidium bromide, as well as putrescine, cadaverine, and spermidine—three polyamines produced by E. coli—are functional ligands of AcrR. We found that ethidium bromide and polyamines bind to AcrR both *in vitro* and *in vivo*, which prevents the binding of AcrR to the *acrAB* promoter and induces the expression of the AcrAB-TolC multidrug efflux pump. Moreover, this study also shows that, besides regulating *acrAB*, AcrR contributes to coping with excess polyamines by directly regulating the expression of the MdtJI inner membrane spermidine efflux pump and the putrescine degradation enzyme PuuA.

## RESULTS

### The AcrR repressor is regulated *in vitro* and *in vivo* by ethidium bromide.

**FIG 1:**
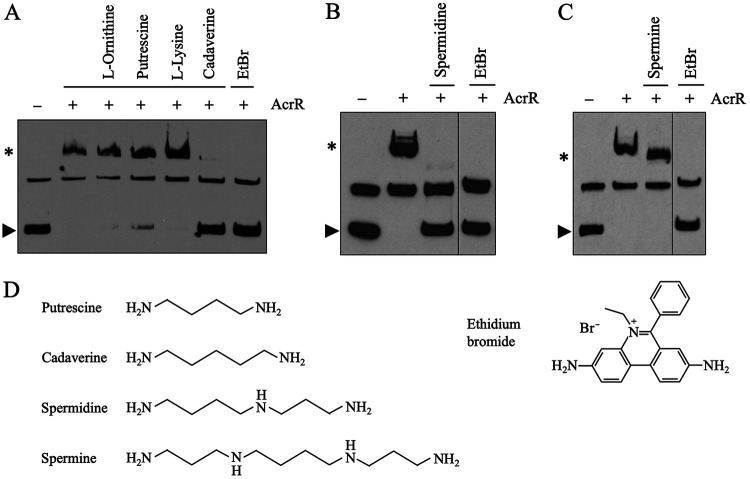
Ethidium bromide and the polyamines putrescine, cadaverine and spermidine prevent binding of AcrR to the *acrAB* promoter. (A to C) EMSAs with biotin-labeled *acrAB* promoter (*acrAB*p), purified AcrR, and candidate ligands were performed to identify ligands capable of binding to AcrR and preventing its binding to *acrAB*p *in vitro*. The results shown are representative of at least three independent assays. From left to right, lane 1 shows *acrAB*p alone, the bottom band (indicated with an arrow) corresponds to the expected size of the *acrAB*p (179 bp), and the top band corresponds to an artifact DNA aggregate observed even when the 179-bp *acrAB*p band was gel purified prior to biotin labeling; lane 2 shows *acrAB*p in the presence of AcrR; binding of AcrR caused a clear shift of the 179-bp *acrAB*p band as expected (indicated with an asterisk). (A) Lanes 3 to 7 show binding reactions with *acrAB*p and AcrR in the presence of 10 mM l-ornithine, 10 mM putrescine, 10 mM l-lysine, 10 mM cadaverine, and 1 mM ethidium bromide (EtBr), respectively. The polyamines putrescine (partially) and cadaverine, and the antimicrobial EtBr, but not the amino acids lysine or ornithine, prevented AcrR binding to *acrAB*p as observed by a lack of shift. (B) Lanes 3 to 4 show binding reactions with *acrAB*p and AcrR in the presence of 5 mM spermidine or 1 mM EtBr, respectively. Spermidine prevented AcrR binding to *acrAB*p as observed by a lack of shift. (C) Lanes 3 to 4 show binding reactions with *acrAB*p and AcrR in the presence of 5 mM spermine or 1 mM EtBr, respectively. Spermine did not prevent AcrR binding to *acrAB*p as observed by the occurrence of a shift. (B and C) Gels split for labeling purposes. We found 10 mM spermidine or spermine to interfere with the assay, thus, those lanes are not included. (D) Chemical structures of ethidium bromide and the polyamines putrescine, cadaverine, spermidine, and spermine.

Based on previous findings (11, 12, 23, 25, 27), we hypothesized that the transcriptional repressor AcrR regulates the expression of the AcrAB-TolC multidrug efflux pump in response to both exogenous toxic compounds and endogenous cellular metabolites. Of the three antimicrobials identified by Su et al. (25) to be capable of binding to AcrR in fluorescence polarization studies, we chose ethidium bromide for further studies because it was the one with the strongest binding affinity to AcrR (25) and has been predicted to fit in the AcrR ligand-binding cavity (23). Using electrophoretic mobility shift assays (EMSAs), we were able to confirm that EtBr binds to AcrR *in vitro* and demonstrate for the first time that such binding changes the activity of AcrR and prevents this regulator from binding to the *acrAB* promoter (*acrAB*p) (Fig. 1A).

**FIG 2:**
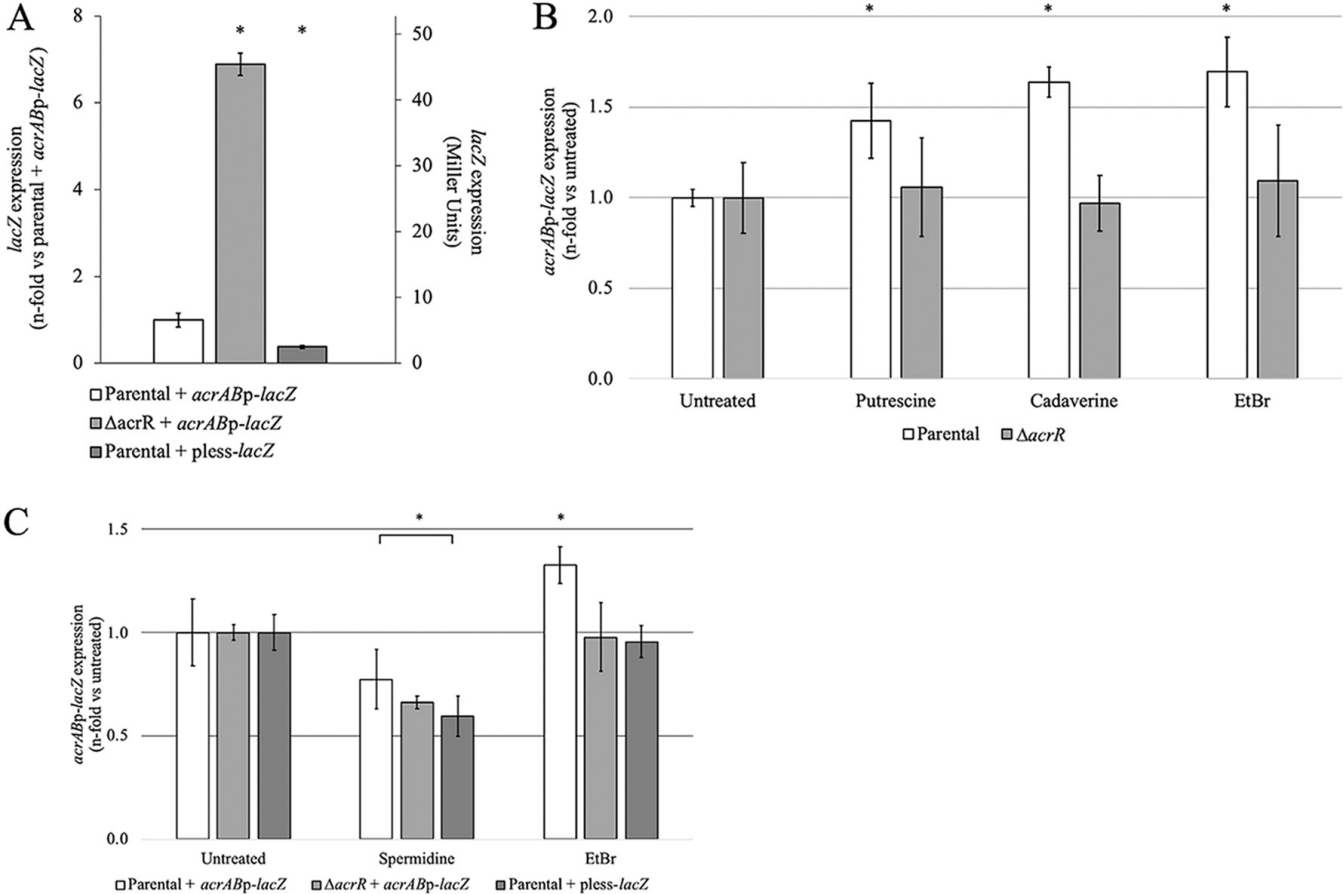
Ethidium bromide, putrescine, and cadaverine activate the *acrAB* promoter (*acrAB*p) *in vivo* in an *acrR*-dependent manner. (A to C) Cultures were grown to mid-exponential phase in LB prior to adding water (untreated), 5 mM polyamine, or 100 μM ethidium bromide (EtBr). After a 1-h incubation, we measured the expression of *acrAB* using an *acrAB*p-*lacZ* fusion. The white bars represent the parental (*acrR*^+^) *acrAB*p-*lacZ* strain. The light gray bars represent the *acrR-*deleted (Δ*acrR*) *acrAB*p-*lacZ* strain. The dark gray bars represent the promoterless (pless) *lacZ* parental strain. All experiments were performed using three to five biological replicates, each one with two technical replicates. The data are presented as average ± SEM (*n *= 3 to 5) and are shown as the *n-*fold change in *acrAB*p-*lacZ* expression versus the parental *acrAB*p-*lacZ* strain (A, left scale) and as raw Miller units (A, right scale) or as the *n-*fold change in *acrAB*p-*lacZ* expression compared to the untreated for each strain (B and C). Statistically significant differences between strains (A) or between the untreated and each treatment (B and C) are indicated by an asterisk (*P < *0.05). (A) *acrAB*p-*lacZ* expression was greatly increased in the Δ*acrR* strain, as previously observed (11, 16). (B) EtBr, putrescine, and cadaverine induced the *acrAB* promoter in the parental but not the Δ*acrR* strain, as would be expected for ligands that bind to and inactivate AcrR. (C) At the concentration tested, spermidine did not induce the *acrAB* promoter. Such lack of induction may be related to the toxicity of this compound in E. coli as suggested by the significant decrease in β-galactosidase expression/activity observed in all three strains studied.

We next studied the effect of EtBr on AcrR and *acrAB* expression *in vivo* using an *acrAB*p-*lacZ* reporter. In agreement with previous findings (11, 16), the *acrAB* promoter was greatly induced in the strain deleted for the AcrR repressor gene (Δ*acrR*) compared to the parental strain (Fig. 2A). Addition of EtBr to the growth medium induced the *acrAB* promoter in the parental, but not the Δ*acrR* strain (Fig. 2B), in agreement with the binding of EtBr to AcrR and subsequent prevention of AcrR binding to *acrAB*p observed by EMSA (Fig. 1A). Of note, lack of induction of *acrAB*p by EtBr in the Δ*acrR* mutant indicates that EtBr regulates this promoter via AcrR and was not the result of *acrAB* expression already being maximal in this mutant because further activation of *acrAB*p in this mutant by 4% ethanol or 0.5 M NaCl stress, or in stationary phase, has been observed before (16).

This finding is also consistent with a previous report of EtBr increasing *acrAB* expression in Edwardsiella tarda, which was hypothesized to occur via AcrR (28). An *in vivo* and AcrR-dependent activation of *acrAB* expression in E. coli or other bacteria by EtBr has not been reported before and confirms EtBr as a functional exogenous ligand of AcrR. Therefore, EtBr was subsequently used as positive control in our search for cellular metabolites that could act as endogenously produced ligands of AcrR, which was the main goal of this work.

### The AcrR repressor is regulated *in vitro* and *in vivo* by polyamines.

We next used EMSA to identify cellular metabolites that bind to AcrR and prevent or reduce its binding to the *acrAB* promoter (Fig. 1). The metabolites tested included the amino acids l-lysine and l-ornithine, which were previously identified as potential AcrR ligands or AcrAB-TolC substrates using untargeted metabolomics (12), as well as their degradation products putrescine and cadaverine. In the presence of molecules that do not bind to and inactivate AcrR, the mobility of the *acrAB* promoter fragment was retarded due to the formation of the higher-molecular-weight AcrR-*acrAB*p complex, as was the case for l-ornithine and l-lysine (Fig. 1A). However, in the presence of putrescine and cadaverine (the decarboxylation products of l-ornithine and l-lysine, respectively), we discovered that the interaction between AcrR and the *acrAB* promoter was partially (putrescine) or completely (cadaverine) disrupted (Fig. 1A), as we had previously observed for EtBr. Interestingly, all three molecules contain two amino groups at opposite ends of the molecule (Fig. 1D). Overall, these finding suggests that both putrescine and cadaverine are also AcrR ligands.

Given that putrescine and cadaverine are two members of a class of structurally related molecules called polyamines (Fig. 1D) produced by all living beings, we also tested by EMSA two other polyamines, spermidine and spermine, for their ability to bind to and disrupt the binding of AcrR to *acrAB*p (Fig. 1B and C). These two polyamines were tested at a lower concentration (5 mM instead of the 10 mM used for putrescine and cadaverine) because higher concentrations of these compounds were found to interfere with the EMSA. Interestingly, we observed a notable disruption of the interaction between AcrR and *acrAB*p in the presence of spermidine but not spermine, which is a larger molecule (Fig. 1B and C). These findings indicate, for the first time, that cellular metabolites that are endogenously produced by E. coli, the polyamines putrescine, cadaverine, and spermidine, can act as ligands that regulate AcrR function.

To further define the role of these three polyamines in regulating the activity of AcrR, we measured the activity of the *acrAB* promoter *in vivo* when they were added to the growth medium. Both putrescine and cadaverine significantly induced *acrAB*p-*lacZ* expression in the parental, but not the Δ*acrR* strain, which was similar to the effect produced by EtBr (Fig. 2B). As discussed above for EtBr, lack of *acrAB*p induction by polyamines in the Δ*acrR* strain was not the result of *acrAB*p being already maximally activated in this strain but, instead, indicates that polyamines regulate *acrAB*p via AcrR. Moreover, this finding is in agreement with our observation that, like EtBr, these polyamines disrupt binding of AcrR to *acrAB*p in the EMSA (Fig. 1A), which further supports putrescine and cadaverine as newly discovered functional ligands of AcrR. A similar effect was not observed for cells grown in the presence of spermidine (Fig. 2C). In contrast, we observed a suppression of β-galactosidase activity in the parental, Δ*acrR*, and even the promoterless-*lacZ* strains (Fig. 2C), suggesting that spermidine added to the growth medium at the concentration tested may have a suppressive effect on either transcription or translation of the *lacZ* gene or the activity of β-galactosidase enzyme. Therefore, we were unable to ascertain whether spermidine induces the *acrAB* promoter *in vivo* in an AcrR-dependent manner, as we observed for putrescine and cadaverine.

### High concentrations of putrescine, cadaverine, and spermidine are toxic to E. coli.

**FIG 3:**
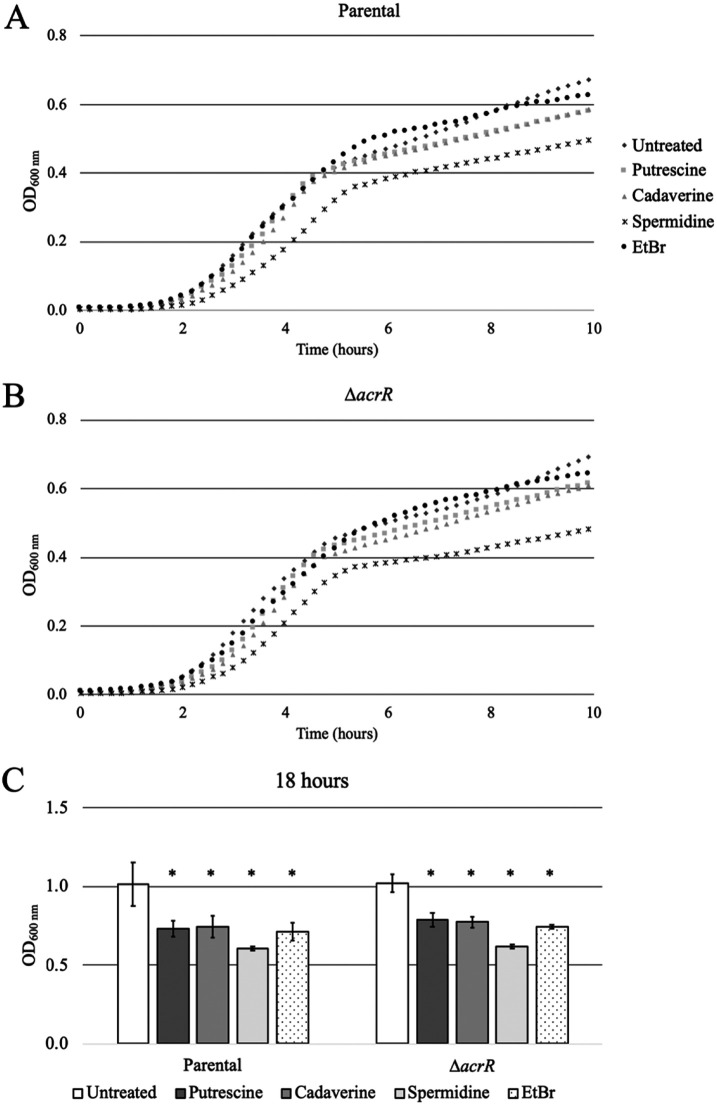
Effect of AcrR ligands on growth of E. coli. Growth curves of the parental (A) and Δ*acrR* (B) strains cultured in LB alone (untreated; dark gray diamond), 5 mM putrescine (light gray square), 5 mM cadaverine (light gray triangle), 5 mM spermidine (black star), and 100 μM ethidium bromide (EtBr; black circle). Only the first 10 h of growth are shown to better display the exponential growth part of the curves. Each growth curve represents the average of three independent biological replicates, each including three technical replicates. The average generation times (in minutes ± the standard deviation; *P* values smaller than 0.1 are also provided) for the parental strain (A) were 27.1 ± 1.9 (untreated), 29.9 ± 3.5 (putrescine), 29.5 ± 3.4 (cadaverine), 31.2 ± 1.5 (spermidine), and 28.7 ± 3.5 (EtBr), and for the Δ*acrR* strain (B), they were 27.4 ± 1.6 (untreated), 30.4 ± 1.4 (putrescine), 32.6 ± 1.8 (cadaverine; *P* = 0.015), 38.8 ± 3.7 (spermidine; *P* = 0.065), and 29.0 ± 1.9 (EtBr). (C) Final OD_600_ yields of the parental and Δ*acrR* strains after 18 h of growth are presented as the average ± SEM (*n* = 3; three biological replicates, each including 3 technical replicates) and statistically significant differences for each strain between the untreated and treated cultures are indicated as an asterisk (*P* < 0.05). For both strains, spermidine caused the most significant decrease in final growth yield (18 h) (C), whereas the other AcrR ligands tested had a smaller but statistically significant decrease on final growth yield (C) at the concentrations tested.

**FIG 4:**
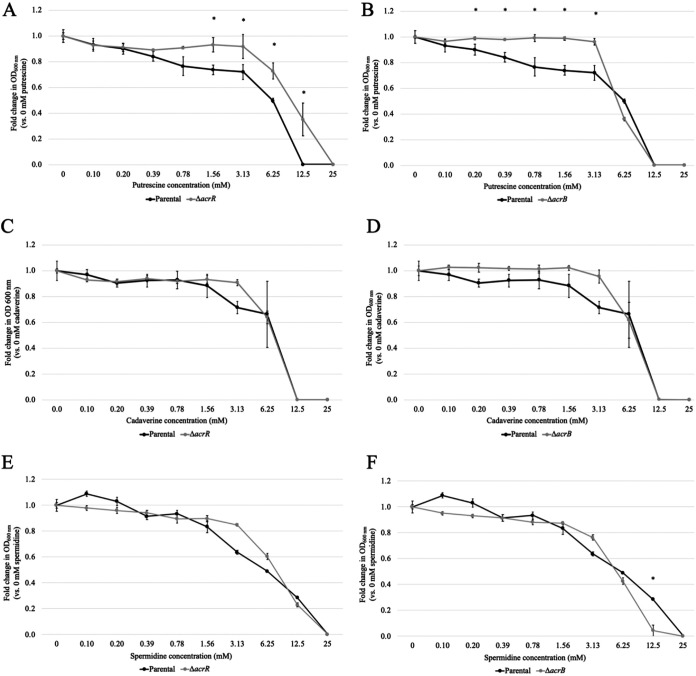
AcrR and the multidrug efflux pump AcrAB-TolC affect the toxicity of putrescine and spermidine. Each panel shows, for each strain, the *n*-fold change in OD_600_ at increasing concentrations of polyamine (putrescine [A and B], cadaverine [C and D], and spermidine [E and F]) compared to the same strain untreated (0-mM) cultures. All experiments were performed using three independent biological replicates, each including three technical replicates. Data are presented as the average ± SEM (*n *= 3). Statistically significant differences between the OD_600_ of the parental and Δ*acrR* (A, C, and E) or the parental and Δ*acrB* (B, D, and F) strains at each concentration of polyamine tested are indicated by an asterisk (*P < *0.05).

To further explore the physiological effects of the three polyamines identified as AcrR ligands, we assessed the ability of the parental and Δ*acrR* strains to grow in their presence (Fig. 3A to C). EtBr is a well-known AcrAB-TolC substrate (3–5) and was included as a control. Because the same concentrations of EtBr or polyamines tested by EMSA (Fig. 1) were too toxic and significantly inhibited growth of both strains, lower (sub-MIC) concentrations were used to measure the effect of these ligands on bacterial growth. At 5 mM, only spermidine produced a small decrease in growth rate during the first 10 h of growth (Fig. 3A and B). However, when we measured the effect of 5 mM polyamines on the final optical density at 600 nm (OD_600_) yield after 18 h of growth, we found that all three polyamines significantly decreased the growth yield compared to the untreated control cells in both strains, with spermidine causing a more substantial growth suppression effect than putrescine or cadaverine (Fig. 3C). These findings suggest that, at 5 mM, exogenously added polyamines are slightly toxic to E. coli. Of note, polyamines were found to be much more toxic in both strains when they were tested at higher concentrations (6.25 to 12.5 mM and greater polyamine concentrations; Fig. 4).

### AcrR and the AcrAB-TolC multidrug efflux pump are involved in mitigating the toxicity of putrescine and spermidine, respectively.

Given that we found that polyamines induced *acrAB* expression in an *acrR*-dependent manner and that they were toxic to E. coli at high concentrations, we next investigated whether AcrR and/or the AcrAB-TolC multidrug efflux pump play a role in mitigating the toxicity caused by the addition of excess polyamines. To address this question, we tested the parental, Δ*acrR*, and Δ*acrB* strains in a broth dilution MIC assay, challenging the strains with putrescine, cadaverine, and spermidine (Fig. 4A to F). We found that deletion of *acrR* did have significant effects on growth in the presence of putrescine (Fig. 4A and B), increasing the MIC of putrescine 2-fold from 12.5 mM to 25 mM. Additionally, deletion of *acrR* and *acrB* also significantly increased the overall OD_600_ yield of cells grown at sub-MICs of putrescine. A similar but more modest increase in overall OD_600_ yield was also found for cadaverine, mainly in the Δ*acrB* mutant (Fig. 4C and D). These findings indicate that the AcrAB-TolC pump plays a very limited or slightly detrimental role in mitigation of putrescine and cadaverine toxicity. Importantly, these findings also suggest that AcrR may repress additional gene(s) involved in detoxification or efflux of putrescine independently from the AcrAB-TolC pump.

Conversely, we found that the MIC of spermidine was affected in the *acrB* mutant but not in the Δ*acrR* mutant, resulting in a small but statistically significant 2-fold decrease from 25 mM to 12.5 mM in Δ*acrB* compared to the parental strain (Fig. 4E and F). The observed small increased sensitivity to spermidine in the *acrB* mutant suggests that efflux by the AcrAB-TolC pump might have some contribution to the removal of excess spermidine.

### AcrR directly regulates genes involved in putrescine detoxification and spermidine efflux.

**FIG 5:**
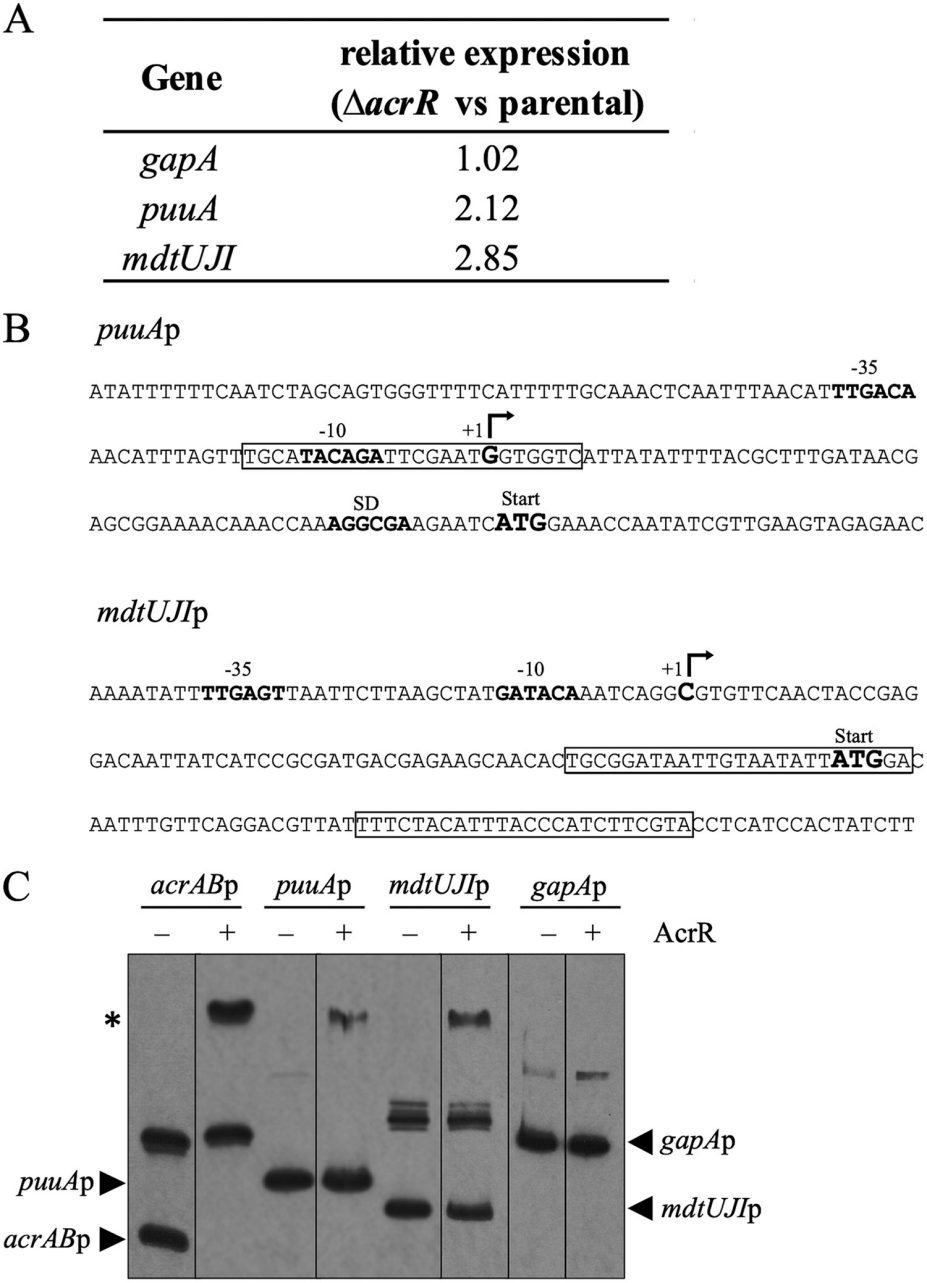
AcrR directly regulates genes involved in polyamine detoxification and efflux. (A) The expression of the polyamine degradation and efflux genes/operons *puuA* and *mdtUJI* was found to be increased *in vivo* in the Δ*acrR* compared to the parental strain in the microarray data from Kim et al. (27). (B) Sequence of the promoter regions of the E. coli glutamate-putrescine ligase gene *puuA* and the spermidine efflux pump operon *mdtUJI*. Predicted AcrR-binding sites are indicated with a box. The −10 and −35 regions, transcriptional start site, Shine-Dalgarno sequence, and translational start site are highlighted in bold lettering. A predicted AcrR-binding site with 9 mismatches and 2 sites with 10 mismatches compared to the AcrR-binding site in the *acrAB* promoter (25) were computationally identified in the *puuA and mdtIUJI* promoters, respectively. This number of mismatches compared to the AcrR-binding site in the *acrAB* promoter is similar to the number of mismatches found for the AcrR-binding sites in the *soxRS* (10 mismatches) and *marRAB* (11 mismatches) promoter regions, both found to be directly regulated by AcrR by Lee et al. (21). (C) EMSA showing *in vitro* binding of purified AcrR to the *acrAB* (positive control), *puuA*, and *mdtUJI* promoters (*acrAB*p, *puuA*p, and *mdtUJI*p, respectively), but not to the *gapA* promoter (*gapA*p, negative control). Gels were split for labeling purposes: both 0.4 and 1 μM AcrR were tested; to save space, only results for 1 μM AcrR are shown, given that no shifts were observed at 0.4 μM for promoters other than *acrAB*p. The results shown are representative of at least three independent assays. The bands corresponding to the expected size of the tested promoters are indicated with arrows on the left (*acrAB*p and *puuA*p) or right (*mdtUJI*p and *gapA*p) sides of the figure. The higher-molecular-weight bands corresponding to the shifted AcrR-promoter complexes for *acrAB*p, *puuA*p, and *mdtUJI*p are indicated with an asterisk on the left side of the figure. Lane 1, *acrAB*p DNA alone (the lower band corresponds to the expected size of the DNA fragment tested); lane 2, *acrAB*p plus AcrR, a clear shift of the lower band observed in line 1 occurred, as expected for binding of AcrR; lane 3, *puuA*p DNA alone; lane 4, *puuA*p plus AcrR, where a clear shift was observed as expected for binding of AcrR, in agreement with the presence of a predicted AcrR-binding site in this promoter (B) and the increased expression of *puuA* previously found in the Δ*acrR* strain (A) (27); lane 5, *mdtUJI*p DNA alone (the bottom band corresponds to the expected size of the DNA fragment tested); lane 6, *mdtUJI*p plus AcrR, where a partial shift was observed as expected for binding of AcrR, in agreement with the presence of a predicted AcrR-binding site in this promoter (B) and the increased expression of *mdtUJI* previously found in the Δ*acrR* compared to the parental strain (A) (27); lane 7, *gapA*p DNA alone; lane 8, *gapA*p plus AcrR, negative control where no shift was observed as expected for the absence of AcrR-binding sites in *gapA*p (not shown) and unchanged *gapA* expression in the Δ*acrR* strain (A) (27).

We next studied the role that AcrR may play, independent from regulating AcrAB-TolC expression, in regulating genes involved in polyamine detoxification or efflux. We first analyzed a previously published microarray (27) and identified two polyamine-related candidates that were upregulated 2- to 3-fold in the Δ*acrR* compared to the parental strain (Fig. 5A), thus indicating that they are repressed by AcrR *in vivo*. These candidates were the *puuA* gene, which encodes a glutamate-putrescine ligase enzyme that catalyzes the first step in the primary putrescine degradation pathway of E. coli (29), and the *mdtUJI* operon, which encodes the MdtJI inner membrane small multidrug resistance (SMR) family spermidine efflux pump (30).

To determine whether regulation of *puuA* and *mdtUJI* by AcrR is direct, we first computationally examined their promoters using the known sequence of the AcrR-binding site in the *acrAB* promoter (25). We found that both promoters contained one and two predicted AcrR-binding sites, respectively (Fig. 5B). This finding suggests that the regulation of both candidates by AcrR is direct. To test this hypothesis, we next used EMSA to determine whether AcrR could directly bind to the *puuA* and *mdtUJI* promoters. For our positive control, we used the *acrAB* promoter. As the negative control, we used a *gapA* promoter fragment because the expression of *gapA* was found not to be altered in the Δ*acrR* mutant (27) and because its promoter region contained no predicted AcrR-binding sites in our computational analysis (not shown). As expected, we found that AcrR directly bound to the *acrAB* but not the *gapA* promoter (Fig. 5C). Interestingly, we also found that AcrR directly bound to and shifted the *puuA* and *mdtUJI* promoter fragments (Fig. 5C). This finding indicates that both *puuA* and *mdtUJI* are newly discovered direct targets of AcrR, and it provides a rationale for the role of AcrR in detoxification and efflux of polyamines, independent from its role in regulating the expression of the AcrAB-TolC pump.

## DISCUSSION

Multidrug efflux pumps are major contributors to the problem of antibiotic resistance and play a major role in bacterial physiological processes such as metabolism and motility (3–5, 8–15). Therefore, it is essential to better understand their function and regulation. Here, we studied the function of the multidrug efflux pump transcriptional repressor AcrR of E. coli, the major regulator controlling the expression of the AcrAB-TolC pump (11, 16). Despite its importance, the ligands that control the activity of AcrR remain unknown, which hinders our understanding of the biological role of this regulator. The findings from the manuscript contribute to addressing this question by showing that the activity of AcrR is controlled *in vitro* and *in vivo* by both exogenous antimicrobial ligands (ethidium bromide) and endogenous/exogenous ligands (polyamines). Moreover, this study reveals that AcrR contributes to coping with toxicity caused by excess polyamines by regulating the expression of AcrAB-TolC and two newly discovered target genes/operons involved in detoxification and efflux of polyamines.

To identify ligands that control the activity of AcrR, we first studied the antimicrobial ethidium bromide (EtBr), a well-known AcrAB-TolC substrate (3–5). We selected EtBr because it showed the highest binding affinity for AcrR in prior fluorescence polarization assays (25) and was later found to fit in the ligand cavity of AcrR by docking analysis (23). Using EMSA and gene expression experiments, we found that binding of EtBr to AcrR indeed prevented binding of this regulator to the *acrAB* promoter both *in vitro* and *in vivo*, which resulted in an *acrR*-dependent induction of *acrAB* expression when EtBr was added to cultures (Fig. 1A and Fig. 2B). These findings show, for the first time, that EtBr is a functional ligand that controls the activity of AcrR.

After finding that the activity of AcrR is regulated by the exogenous ligand EtBr, we next studied whether this regulator also responds to endogenous ligands, which was the main goal of this work. Based on prior studies described more in detail in the introduction (11, 12, 23, 25, 27), we hypothesized that AcrR acts as a sensor of endogenous cellular metabolites that are either substrates of the AcrAB-TolC pump or their metabolic precursors or derivatives. Notably, several of the cellular metabolites showing the most significantly altered levels in efflux mutants in our prior metabolomics study were amino acids and their biosynthesis or degradation intermediates (12). Of them, l-lysine was among the most significantly accumulated metabolites in strains deleted for *acrB* or *tolC* (12). Thus, we next investigated whether the amino acids l-lysine and l-ornithine (which is structurally related to l-lysine), or their polyamine derivatives putrescine, cadaverine, spermidine, and spermine, were involved in regulating the activity of AcrR.

Using EMSA, we found that while lysine and ornithine had no effect, the polyamines putrescine, cadaverine, and spermidine (but not spermine) acted as endogenous ligands of AcrR (Fig. 1A and C). As we had observed for EtBr, we found that cadaverine and putrescine bind to and prevent AcrR binding to the *acrAB* promoter both *in vitro* and *in vivo*, which resulted in an *acrR*-dependent induction of *acrAB* expression when they were added to E. coli cultures (Fig. 1A and Fig. 2B). Spermidine also regulated the activity of AcrR *in vitro*, although its *in vivo* role could not be ascertained because of its overall suppressive effect in our assay (Fig. 1C and Fig. 2C). These findings indicate that, besides the exogenous antimicrobial EtBr, AcrR also responds endogenous ligands, i.e., polyamines, which has not been reported before to the best our knowledge.

Interestingly, both EtBr and the three polyamines identified as functional AcrR ligands share multiple functional, structural, and physical-chemical features, including their toxicity at high concentrations, a 52- to-64 Å topological polar surface area, a hydrogen bond donor and acceptor count of 2 to 3, and, especially, the presence of two amino groups at opposite ends of the molecule (Fig. 1D) (https://pubchem.ncbi.nlm.nih.gov, compounds 14710, 1045, 273, and 1102). These findings support a central role of AcrR in responding to both exogenous and endogenous positively charged toxic molecules, which ultimately would trigger the upregulation of the AcrAB-TolC pump and, as discussed below, of polyamine efflux and detoxification genes in order for cells to cope with these toxic molecules.

Polyamines are present at millimolar levels in cells, where they interact with a broad range of macromolecules, including DNA, and have pleiotropic effects (31–35). It is important noticing that, although polyamines are known to interact with DNA because of their polycationic nature, their role in preventing binding of AcrR to the *acrAB* promoter was found to be specific and to occur at concentrations similar to those found in cells, as well as both *in vitro* and *in vivo*. In particular, the finding that putrescine, cadaverine, and spermidine, but not spermine (which is chemically and structurally similar but a longer molecule than spermidine) or l-lysine (which has two positive charged groups in its structure), prevented AcrR binding to the *acrAB* promoter strongly supports that the effect observed for putrescine, cadaverine, and spermidine was caused by their specific interaction with AcrR. Interestingly, the three polyamines found here to be ligands of AcrR, putrescine, cadaverine, and spermidine are synthesized by E. coli (32, 33), whereas spermine, which is produced by humans but not E. coli, was the only polyamine that did not function as an AcrR ligand. Polyamines increase survival in acid conditions because the amino acid decarboxylation reactions that produce them consume protons, which, together with their ability to decrease outer membrane permeability by blocking the OmpF and OmpC porins, protect cells from acid stress (32, 33). However, excess polyamines can be toxic because they inhibit protein synthesis and decrease cell growth at neutral and, especially, basic pH (32–34).

Given their role as AcrR ligands, we decided to study the role of AcrR and AcrAB-TolC in coping with excess polyamines using growth and MIC assays (Fig. 3 and 4). Our most significant findings were that deletion of *acrR* made cells 2-fold more resistant to putrescine than the parental strain, whereas deletion of *acrB* made cells 2-fold more sensitive to spermidine (Fig. 4). First, these findings indicate that AcrAB-TolC contributes to mitigating the toxicity of spermidine but not putrescine or cadaverine. Second, these findings also suggest that AcrR plays a role in repressing genes important for resistance to excess putrescine, which is independent from its role as a repressor of the *acrAB* operon. Of note, it is possible that the role of AcrR and/or AcrAB-TolC in coping with excess polyamines may have been larger when measured at basic pH, given the greater toxicity of polyamines at high pH (34). Moreover, given the pleiotropic effects of polyamines (31–35), it is also possible that polyamines may be effluxed for reasons other than coping with their toxicity.

To further analyze the role of AcrR in coping with excess polyamines independent from its role as an *acrAB* repressor, we searched for polyamine-related genes that may be regulated by AcrR. We identified two candidates, the *puuA* gene, which encodes a glutamate-putrescine ligase enzyme responsible for the first step in the main putrescine degradation pathway of E. coli (pathway II) (29), and the *mdtUJI* operon, which encodes the MdtJI SMR family inner membrane efflux pump involved in efflux of excess spermidine (30). Both *puuA* and *mdtUJI* were significantly upregulated *in vivo* in the Δ*acrR* mutant compared to the parental strain in a previously published microarray (27), which indicates that they are regulated by the AcrR repressor. Thus, we next studied whether such regulation by AcrR is direct. Computational analysis of both promoters revealed that they contain predicted AcrR-binding sites, and our EMSAs revealed that AcrR specifically binds to both promoters (Fig. 5B and C). These findings indicate that AcrR is indeed a direct regulator of both polyamine detoxification/efflux promoters.

Overall, our findings for AcrAB-TolC and AcrR in relationship with polyamines are of significance for several reasons. First, AcrR seems to control efflux of excess spermidine both across the inner membrane via MdtJI and across the outer membrane via AcrAB-TolC, which suggests that both transporters may act synergistically or in tandem. It is interesting that despite these roles, the Δ*acrR* mutant did not show an increased MIC for spermidine compared to the parental strain. Several reasons may explain this finding. It is possible that because of its 2-fold dilution nature, the MIC assay was not sensitive enough and that differences might have been observed when testing spermidine concentrations between 12.5 and 25 mM. It is also possible that the effect of inactivating *acrR* might have been observed in experiments performed at basic pH, given the greater toxicity of polyamines at high pH discussed above. Another possibility is that inactivation of *acrR* produces other regulatory changes in the cell that counteract the protective effect of having increased *acrAB* and *mdtUJI* expression.

Second, to our knowledge, a role of AcrR in directly regulating a metabolic gene such as *puuA* has not been reported before. This significant finding expands the role of AcrR from being only an efflux-related regulator and raises the question of which other detoxification or metabolic processes are also directly controlled by this regulator.

In conclusion, the manuscript reveals that both exogenous antimicrobials, such as ethidium bromide, and endogenously produced cellular metabolites, such as polyamines, act as functional ligands of AcrR both *in vitro* and *in vivo*. As a result, these ligands induce the expression of the AcrAB-TolC multidrug efflux pump, which, in turn, contributes to the removal of toxic molecules such as EtBr or excess spermidine. In addition, we report that AcrR also contributes to coping with excess polyamines independently from AcrAB-TolC by directly regulating the expression of the MdtJI inner membrane spermidine efflux pump and the *puuA* putrescine degradation gene. Overall, these findings significantly advance our understanding of the biological role of AcrR by uncovering the ligands that control the function of AcrR and identifying novel efflux and metabolic genes directly controlled by this regulator.

## MATERIALS AND METHODS

### Bacterial cultures.

Cultures of Escherichia coli strains for growth curves, MIC, and gene expression experiments were grown at 37°C with agitation (200 rpm) in lysogeny broth (LB; 5 g/L yeast extract, 10 g/L tryptone, and 10 g/L NaCl). E. coli strains that carry the pNN608 or pNN387 plasmids were cultured in LB medium supplemented with 20 μg/mL of chloramphenicol. Strain DH7293, which contains the pET21(+)-*acrR* plasmid, was cultured in LB supplemented with 100 μg/mL of ampicillin.

### Strains, plasmids, and genetic procedures.

The bacterial strains and plasmids used in this study are listed in Table 1. Plasmid pET21(+)-*acrR* was constructed by PCR amplification of the *acrR* gene from the parental E. coli BW25113 strain using the primers acrRF1-BamHI (5′-CCAGGATCCAATAATTTGTACTTAGAAGGAGATATACCATGGCACGAAAAACCAAACAA-3′) and acrRR1-XhoI (5′-GATCCTCGAGTTCGTTAGTGGCAGGATTACGAAG-3′). After amplification, the PCR product was gel purified, digested with BamHI and XhoI (NEB, Ipswich, MA), and ligated into pET21(+) linearized with the same enzymes generating plasmid pET21(+)-*acrR*. This plasmid, which contains an IPTG (isopropyl-β-d-thiogalactopyranoside)-inducible promoter, a ribosome binding site, and the *acrR* gene with a 6×His C-terminal tag, was then electroporated into E. coli BL21(DE3) to generate strain DH7293. Correct cloning of *acrR* into pET21(+)-*acrR* was confirmed by PCR followed by Sanger sequencing at Laragen Inc. (Culver City, CA). Overexpression of AcrR in strain DH7293 after addition of 100 μM IPTG was confirmed by Coomassie staining.

**TABLE 1:** Bacterial strains and plasmids used in this study

Strain or plasmid	Description	Reference or source(s)
E. coli strains		
BL21(DE3)	F^–^ *ompT gal dcm lon hsdS_B_* (*r_B_*^–^ *m_B_*^–^) λ(DE3 [*lacI lacUV5*-*T7p07 ind1 sam7 nin5*]) [*malB*^+^]_K-12_(λ^s^)	40
BW25113	(Parental) F^–^ λ^–^ Δ*(araD-araB)567* Δ*lacZ4787*(::*rrnB-3*) *rph-1* Δ*(rhaD-rhaB)568 hsdR514*	CGSC, Keio collection, 37
DH7311	BW25113 Δ*acrR*	This study
CR6000	BW25113 + pNN608	11
CR7011	BW25113 Δ*acrR* + pNN608	11
DH7169	BW25113 + pNN387	This study
DH7293	BL21 + pET21(+)-*acrR*	This study
Plasmids		
pET21(+)	AmpR, MCS, *col* E1 *ori*, *f1 ori*, T7-lac promoter	Novagen, WI, USA
pET21(+)-*acrR*	pET21(+) derivative, AmpR, IPTG-inducible vector to overexpress AcrR with a 6×His C-terminal tag	This study
pCP20	Plasmid for excision of kan markers by FLP-mediated site recombination, AmpR, ChlR	36
pNN387	Single copy, promoterless *lacZ*, ChlR	16
pNN608	Single copy, *acrAB*p-*lacZ*, ChlR	16

E. coli DH7311 (BW25113 Δ*acrR*) was constructed by using the pCP20 plasmid as previously described (36) to remove the *kanR* cassette from the Keio collection strain JW0453 (BW25113 *acrR*::*kan* [37]). Correct excision of the *kanR* cassette was confirmed by PCR amplification and Sanger sequencing using primers flanking the *acrR* gene, as previously described (11).

### AcrR purification.

To purify AcrR, an overnight culture of E. coli DH7293 was grown in LB medium containing 100 μg/mL ampicillin (LB-amp). The overnight culture was diluted 1:50 into 500 mL of LB-amp, which was incubated with shaking for 2.5 h before IPTG was added to a final concentration of 100 μM to induce AcrR expression. After the culture was incubated for another 3 h, cells were pelleted by centrifugation at 10,000 × *g* for 10 min at 4°C, and the pellets were resuspended in NH buffer (200 mM NaCl, 20 mM HEPES, pH 7.2) supplemented with EDTA-free protease inhibitors (PIs) (Pierce, Waltham, MA). Cells were subjected to a freeze-thaw cycle before being treated with DNase I (2 U/mL) and lysozyme (10 mg/mL in 50 mM Tris-HCl, pH 7.5) for 15 min at room temperature. Next, cells were lysed using a French pressure cell press (SLM Instruments, Inc.) at 17,000 lb/in^2^. Lysates were centrifuged for 5 min at 15,000 × *g* at 4°C to remove unbroken cells and large aggregates. The crude lysate was filtered through a 0.22-μm filter before being applied to a nitriloacetic acid (NTA)-Sepharose column equilibrated in NH buffer. The column was washed twice with 5 volumes of column wash buffer (200 mM NaCl, 20 mM HEPES, pH 7.2, 50 mM imidazole, and PIs), and then the AcrR protein was eluted in 5 volumes of elution buffer (200 mM NaCl, 20 mM HEPES, pH 7.2, 5% glycerol, 400 mM imidazole, and PIs). Eluted AcrR protein was stored at 4°C in elution buffer.

### Electrophoretic mobility shift assays.

For electrophoretic mobility shift assays (EMSAs), purified AcrR was first exchanged four times into NHG buffer (200 mM NaCl, 20 mM HEPES, pH 7.2, 5% glycerol, and PIs) by serial concentration and dilution (5:1 and 1:5, respectively), which diluted away imidazole to a final concentration of 0.8 mM. Once exchanged, the protein concentration was quantified by Coomassie Bradford assay (Thermo Fisher Scientific, Waltham, MA), and then AcrR was diluted to a final concentration of 4 μM in NHG buffer.

All DNA-promoter fragments were generated by PCR using E. coli BW25113 as the source of template DNA. The 179-bp *acrAB* promoter fragment was generated using the forward primer, pacrABup (5′-CTTGCGCTTCTTGTTTGG), and the reverse primer, pacrABdn (5′-TGTTCATATGTAAACCTCGAG). The 226-bp *puuA* promoter fragment was generated using the forward primer, puuApF2 (5′-GTGGACTAAATTATCGCC), and the reverse primer, puuApR2 (5′-GTTCTCTACTTCAACGAT). The 286-bp *mdtUJI* promoter fragment was generated using the forward primer, mdtUJIpF1 (5′-CGCTCAGGTAAAGAAGTG), and the reverse primer, mdtUJIpR1 (5′-CAAAGTAAAGTGGTCAAGC). The 249-bp *gapA* promoter fragment was generated with the forward primer, gapAF2 (5′GCTGCACCTAAATCGTGATGA), and the reverse primer, gapAR1 (5′-CTTTGATAGTCATATATTCCACCAGCTA). The PCR-generated fragments were column purified or gel extracted (Zymo Research, Irvine, CA) before adding a 3′-biotin label using the Pierce biotin 3′-end DNA labeling kit (Thermo Fisher Scientific, Waltham, MA). The biotinylated DNA was diluted in water to a final concentration of 50 nM.

Ethidium bromide (EtBr) was prepared in sterile DNA-grade water (Fisher Scientific, Hampton, NH) as a 10-mM stock solution. The amino acid and polyamine ligand stock solutions were all prepared in sterile DNA-grade water at 100 mM.

EMSA was performed using the LightShift chemiluminescent EMSA kit from Thermo Fisher Scientific (Waltham, MA). To study potential AcrR ligands, 20-μL EMSA reaction mixtures were set up as follows: 1× binding buffer (10 mM Tris-HCl, pH 7.5, 50 mM KCl, and 1 mM dithiothreitol [DTT]), 5 mM MgCl_2_, 1 μg poly(dI-dC), 0.05% NP-40, 5% glycerol, 25 fmol biotin-*acrAB*p DNA, 400 nM AcrR (except in the DNA-only control reaction), and potential ligand (except for the DNA-only and DNA-plus-AcrR-only reactions) at a final concentration of 1 mM (EtBr), 10 mM (l-ornithine, l-Lysine, putrescine, and cadaverine), or 5 mM (spermidine and spermine). The potential ligands were first permitted to incubate in the reaction mixtures with purified AcrR for 5 min at room temperature (RT) prior to the addition of biotin-*acrAB*p DNA. The final reaction mixtures were allowed to incubate at RT for 20 min before the addition of loading buffer and were then held on ice for 20 min before being loaded into a 10% polyacrylamide DNA gel. After electrophoresis, the biotin-labeled DNA in the gel was transferred to a Biodyne B nylon membrane and probed using the LightShift chemiluminescent EMSA kit streptavidin-horseradish peroxidase (HRP) conjugate as recommended by the manufacturer.

To study binding of AcrR to the *puuA*, *mdtUJI*, and *gapA* promoter fragments, as well as the *acrAB* promoter as positive control, all EMSAs were performed essentially as described above using, for each promoter, DNA-only and DNA plus 0.4 and 1 μM AcrR-only reactions. In these assays, a higher concentration of AcrR (1 μM) than that used in the *acrAB*p-AcrR-ligand assays described above (0.4 μM) was used to increase the sensitivity of the assay in detecting potential binding of AcrR to *puuA*p and *mdtUJI*p. Of note, *gapA*p was added as a negative control to ensure that the assay remained specific, whereas *acrAB*p was used as positive control and for comparison with the shifts observed for 400 nM AcrR.

### β-Galactosidase assays.

The expression of the *acrAB*p*-lacZ* transcriptional fusion found in the single-copy plasmid pNN608 was measured by β-galactosidase assays performed essentially as previously described (11, 38). Briefly, the E. coli strains CR6000 (parental *acrAB*p*-lacZ*), CR7011(Δ*acrR acrAB*p*-lacZ*), and DH7169 (parental that contains the promoterless *lacZ* [pless-*lacZ*] control plasmid pNN387 and was used as a β-galactosidase background control) were grown overnight in LB containing 20 μg/mL of chloramphenicol (LB-chl). The following day, the cultures were subcultured 1:1,000 into LB-chl and allowed to grow at 37°C with agitation for 3 h. EtBr or polyamines (prepared as described above) were then added at a final concentration of 100 μM or 5 mM, respectively, and cells were incubated for an additional hour, concentrated 20×, and their β-galactosidase activity measured.

### Growth curve experiments.

To determine the effect of EtBr, putrescine, cadaverine, and spermidine on E. coli growth in the presence and absence of *acrR*, we prepared overnight cultures in LB of strains BW25113 (parental) and DH7311 (Δ*acrR*). These cultures were then subcultured 1:1,000 into 96-well microplates containing 100 μL of LB (untreated) or LB supplemented with 100 μM EtBr or 5 mM polyamine. We then measured the OD_600_ of each well every 4 min for 18 h, with 15 s of agitation prior to each read, using a Victor Nivo S5 multimode plate reader (PerkinElmer, Waltham, MA).

### Determination of the MICs of polyamines.

The MICs of putrescine, cadaverine, and spermidine for the parental (BW25113), Δ*acrR* (DH7311), and Δ*acrB* (CR5000) E. coli strains were determined using the standard broth microdilution method as previously described (39), with the following modifications. LB was chosen instead of cation-adjusted Mueller-Hinton broth (MHB) because MHB is not usually recommended for testing polycationic molecules (39) and to be able to directly relate our MIC findings to our gene expression and growth experiment results, which were all performed using LB. Briefly, all MIC assays were conducted in 96-well microplates using 2-fold serial dilutions of the tested compounds in 100 μL LB. Wells were inoculated using cells that were first grown overnight in LB and then washed in sterile 0.85% saline prior to standardizing their OD_600_ to a 0.5 McFarland standard. OD_600_ measurements after 18 h of growth were made using a Victor Nivo S5 multimode plate reader, and the MIC was determined as the compound concentration that produced a 95% reduction or greater in growth compared to the untreated.

### Identification of AcrR-regulated polyamine genes.

Candidate polyamine metabolism or transport genes regulated by the AcrR repressor were first selected by identifying those polyamine-related genes significantly upregulated in the Δ*acrR* mutant compared to the parental strain in a previously published microarray (27). In this search, the *puuA* putrescine metabolism gene and the *mdtUJI* spermidine export operon were both found to be regulated by AcrR. To determine if such regulation may be direct, we searched for predicted AcrR-binding sites within and in the 1,000-bp upstream promoter regions of *puuA* and *mdtUJI* using the 24-bp AcrR-binding site in the *acrAB* promoter (5′-TACATACATTTGTGAATGTATGTA-3′) (25) and the Colibri search tool (http://genolist.pasteur.fr/Colibri/) set at a cutoff of 11 mismatches.

### Statistical analysis.

Statistically significant differences in β-galactosidase, growth curve, and MIC experiments were determined using the *t* test (two independent samples with equal variance, two-tailed distribution) using Microsoft Excel 2019 software.

## References

[1] O’Neill J. 2014. Antimicrobial resistance: tackling a crisis for the health and wealth of nations. The review on antimicrobial resistance chaired by Jim O'Neill. https://amr-review.org/sites/default/files/AMR%20Review%20Paper%20-%20Tackling%20a%20crisis%20for%20the%20health%20and%20wealth%20of%20nations_1.pdf. Accessed 15 September 2022.

[2] Centers for Disease Control and Prevention. 2019. Antibiotic resistance threats in the United States, 2019. https://stacks.cdc.gov/view/cdc/82532. Accessed 15 September 2022.

[3] Li XZ, Plésiat P, Nikaido H. 2015. The challenge of efflux-mediated antibiotic resistance in Gram-negative bacteria. Clin Microbiol Rev 28:337–418. doi:10.1128/CMR.00117-14. 25788514 PMC4402952

[4] Li X, Elkins CA, Zgurskaya HI. 2016. Efflux-mediated antimicrobial resistance in bacteria: mechanisms, regulation and clinical implications. Springer Berlin Heidelberg, New York, NY.

[5] Du D, Wang-Kan X, Neuberger A, van Veen HW, Pos KM, Piddock LJV, Luisi BF. 2018. Multidrug efflux pumps: structure, function and regulation. Nat Rev Microbiol 16:523–539. doi:10.1038/s41579-018-0048-6. 30002505

[6] Nolivos S, Cayron J, Dedieu A, Page A, Delolme F, Lesterlin C. 2019. Role of AcrAB-TolC multidrug efflux pump in drug-resistance acquisition by plasmid transfer. Science 364:778–782. doi:10.1126/science.aav6390. 31123134

[7] Langevin AM, El Meouche I, Dunlop MJ. 2020. Mapping the role of AcrAB-TolC efflux pumps in the evolution of antibiotic resistance reveals near-MIC treatments facilitate resistance acquisition. mSphere 5:e01056-20. doi:10.1128/mSphere.01056-20. 33328350 PMC7771234

[8] Helling RB, Janes BK, Kimball H, Tran T, Bundesmann M, Check P, Phelan D, Miller C. 2002. Toxic waste disposal in *Escherichia coli*. J Bacteriol 184:3699–3703. doi:10.1128/JB.184.13.3699-3703.2002. 12057966 PMC135154

[9] Rosner JL, Martin RG. 2009. An excretory function for the *Escherichia coli* outer membrane pore TolC: upregulation of *marA* and *soxS* transcription and Rob activity due to metabolites accumulated in *tolC* mutants. J Bacteriol 191:5283–5292. doi:10.1128/JB.00507-09. 19502391 PMC2725600

[10] Zgurskaya HI, Krishnamoorthy G, Ntreh A, Lu S. 2011. Mechanism and function of the outer membrane channel TolC in multidrug resistance and physiology of enterobacteria. Front Microbiol 2:189. doi:10.3389/fmicb.2011.00189. 21954395 PMC3174397

[11] Ruiz C, Levy SB. 2014. Regulation of *acrAB* expression by cellular metabolites in *Escherichia coli*. J Antimicrob Chemother 69:390–399. doi:10.1093/jac/dkt352. 24043404 PMC3886929

[12] Cauilan A, Ramos K, Harmon DE, Ruiz C. 2019. Global effect of the AcrAB–TolC multidrug efflux pump of *Escherichia coli* in cell metabolism revealed by untargeted metabolomics. Int J Antimicrob Agents 54:105–107. doi:10.1016/j.ijantimicag.2019.05.015. 31108224

[13] Wang-Kan X, Rodriguez-Blanco G, Southam AD, Winder CL, Dunn WB, Ivens A, Piddock LJV. 2021. Metabolomics reveal potential natural substrates of AcrB in *Escherichia coli* and *Salmonella enterica* serovar Typhimurium. mBio 12:e00109-21. doi:10.1128/mBio.00109-21. 33785633 PMC8092203

[14] Shirshikova TV, Sierra-Bakhshi CG, Kamaletdinova LK, Matrosova LE, Khabipova NN, Evtugyn VG, Khilyas IV, Danilova IV, Mardanova AM, Sharipova MR, Bogomolnaya LM. 2021. The ABC-type efflux pump MacAB is involved in protection of *Serratia marcescens* against aminoglycoside antibiotics, polymyxins, and oxidative stress. mSphere 6:e00033-21. doi:10.1128/mSphere.00033-21. 33692192 PMC8546677

[15] Teelucksingh T, Thompson LK, Zhu S, Kuehfuss NM, Goetz JA, Gilbert SE, MacNair CR, Geddes-McAlister J, Brown ED, Cox G. 2022. A genetic platform to investigate the functions of bacterial drug efflux pumps. Nat Chem Biol doi:10.1038/s41589-022-01119-y. 36065018

[16] Ma D, Alberti M, Lynch C, Nikaido H, Hearst JE. 1996. The local repressor AcrR plays a modulating role in the regulation of *acrAB* genes of *Escherichia coli* by global stress signals. Mol Microbiol 19:101–112. doi:10.1046/j.1365-2958.1996.357881.x. 8821940

[17] Rosenberg EY, Bertenthal D, Nilles ML, Bertrand KP, Nikaido H. 2003. Bile salts and fatty acids induce the expression of *Escherichia coli* AcrAB multidrug efflux pump through their interaction with Rob regulatory protein. Mol Microbiol 48:1609–1619. doi:10.1046/j.1365-2958.2003.03531.x. 12791142

[18] Zhang A, Rosner JL, Martin RG. 2008. Transcriptional activation by MarA, SoxS and Rob of two *tolC* promoters using one binding site: a complex promoter configuration for *tolC* in *Escherichia coli*. Mol Microbiol 69:1450–1455. doi:10.1111/j.1365-2958.2008.06371.x. 18673442 PMC2574956

[19] Ruiz C, Levy SB. 2010. Many chromosomal genes modulate MarA-mediated multidrug resistance in *Escherichia coli*. Antimicrob Agents Chemother 54:2125–2134. doi:10.1128/AAC.01420-09. 20211899 PMC2863627

[20] Martin RG, Rosner JL. 2011. Promoter discrimination at class I MarA regulon promoters mediated by glutamic acid 89 of the MarA transcriptional activator of *Escherichia coli*. J Bacteriol 193:506–515. doi:10.1128/JB.00360-10. 21097628 PMC3019838

[21] Lee JO, Cho KS, Kim OB. 2014. Overproduction of AcrR increases organic solvent tolerance mediated by modulation of SoxS regulon in *Escherichia coli*. Appl Microbiol Biotechnol 98:8763–8773. doi:10.1007/s00253-014-6024-9. 25176444

[22] Ramos JL, Martinez-Bueno M, Molina-Henares AJ, Teran W, Watanabe K, Zhang X, Gallegos MT, Brennan R, Tobes R. 2005. The TetR family of transcriptional repressors. Microbiol Mol Biol Rev 69:326–356. doi:10.1128/MMBR.69.2.326-356.2005. 15944459 PMC1197418

[23] Li M, Gu R, Su CC, Routh MD, Harris KC, Jewell ES, McDermott G, Yu EW. 2007. Crystal structure of the transcriptional regulator AcrR from *Escherichia coli*. J Mol Biol 374:591–603. doi:10.1016/j.jmb.2007.09.064. 17950313 PMC2254304

[24] Cuthbertson L, Nodwell JR. 2013. The TetR family of regulators. Microbiol Mol Biol Rev 77:440–475. doi:10.1128/MMBR.00018-13. 24006471 PMC3811609

[25] Su CC, Rutherford DJ, Yu EW. 2007. Characterization of the multidrug efflux regulator AcrR from *Escherichia coli*. Biochem Biophys Res Commun 361:85–90. doi:10.1016/j.bbrc.2007.06.175. 17644067 PMC2104644

[26] Su CC, Nikaido H, Yu EW. 2007. Ligand-transporter interaction in the AcrB multidrug efflux pump determined by fluorescence polarization assay. FEBS Lett 581:4972–4976. doi:10.1016/j.febslet.2007.09.035. 17910961 PMC2254335

[27] Kim YJ, Im SY, Lee JO, Kim OB. 2016. Potential swimming motility variation by AcrR in *Escherichia coli*. J Microbiol Biotechnol 26:1824–1828. doi:10.4014/jmb.1607.07058. 27558437

[28] Hou JH, Hu YH, Zhang M, Sun L. 2009. Identification and characterization of the AcrR/AcrAB system of a pathogenic *Edwardsiella tarda* strain. J Gen Appl Microbiol 55:191–199. doi:10.2323/jgam.55.191. 19590146

[29] Kurihara S, Oda S, Tsuboi Y, Kim HG, Oshida M, Kumagai H, Suzuki H. 2008. Gamma-glutamylputrescine synthetase in the putrescine utilization pathway of *Escherichia coli* K-12. J Biol Chem 283:19981–19990. doi:10.1074/jbc.M800133200. 18495664

[30] Higashi K, Ishigure H, Demizu R, Uemura T, Nishino K, Yamaguchi A, Kashiwagi K, Igarashi K. 2008. Identification of a spermidine excretion protein complex (MdtJI) in *Escherichia coli*. J Bacteriol 190:872–878. doi:10.1128/JB.01505-07. 18039771 PMC2223573

[31] Chattopadhyay MK, Tabor CW, Tabor H. 2003. Polyamines protect *Escherichia coli* cells from the toxic effect of oxygen. Proc Natl Acad Sci USA 100:2261–2265. doi:10.1073/pnas.2627990100. 12591940 PMC151328

[32] Miller-Fleming L, Olin-Sandoval V, Campbell K, Ralser M. 2015. Remaining mysteries of molecular biology: the role of polyamines in the cell. J Mol Biol 427:3389–3406. doi:10.1016/j.jmb.2015.06.020. 26156863

[33] Reitzer L. 2005. Catabolism of amino acids and related compounds. EcoSal Plus 1. doi:10.1128/ecosalplus.3.4.7. 26443507

[34] Yohannes E, Thurber AE, Wilks JC, Tate DP, Slonczewski JL. 2005. Polyamine stress at high pH in *Escherichia coli* K-12. BMC Microbiol 5:59. doi:10.1186/1471-2180-5-59. 16223443 PMC1274320

[35] Yoshida M, Kashiwagi K, Shigemasa A, Taniguchi S, Yamamoto K, Makinoshima H, Ishihama A, Igarashi K. 2004. A unifying model for the role of polyamines in bacterial cell growth, the polyamine modulon. J Biol Chem 279:46008–46013. doi:10.1074/jbc.M404393200. 15326188

[36] Datsenko KA, Wanner BL. 2000. One-step inactivation of chromosomal genes in *Escherichia coli* K-12 using PCR products. Proc Natl Acad Sci USA 97:6640–6645. doi:10.1073/pnas.120163297. 10829079 PMC18686

[37] Baba T, Ara T, Hasegawa M, Takai Y, Okumura Y, Baba M, Datsenko KA, Tomita M, Wanner BL, Mori H. 2006. Construction of *Escherichia coli* K-12 in-frame, single-gene knockout mutants: the Keio collection. Mol Syst Biol 2:2006.0008. doi:10.1038/msb4100050. PMC1681482 16738554

[38] Ruiz C, McMurry LM, Levy SB. 2008. Role of the multidrug resistance regulator MarA in global regulation of the *hdeAB* acid resistance operon in *Escherichia coli*. J Bacteriol 190:1290–1297. doi:10.1128/JB.01729-07. 18083817 PMC2238188

[39] Wiegand I, Hilpert K, Hancock RE. 2008. Agar and broth dilution methods to determine the minimal inhibitory concentration (MIC) of antimicrobial substances. Nat Protoc 3:163–175. doi:10.1038/nprot.2007.521. 18274517

[40] Studier FW, Rosenberg AH, Dunn JJ, Dubendorff JW. 1990. Use of T7 RNA polymerase to direct expression of cloned genes. Methods Enzymol 185:60–89. doi:10.1016/0076-6879(90)85008-c. 2199796

